# 61. Short- versus prolonged-courses of antimicrobial therapy for patients with uncomplicated *Pseudomonas aeruginosa* bloodstream infection

**DOI:** 10.1093/ofid/ofab466.061

**Published:** 2021-12-04

**Authors:** Moonsuk Bae, Yun-Seo Jeong, Seongman Bae, Min Jae Kim, Yong Pil Chong, Sung-Han Kim, Sang-Oh Lee, Sang-Ho Choi, Yang Soo Kim, Jiwon Jung

**Affiliations:** 1 Pusan National University Yangsan Hospital, Yangsan-si, Kyongsang-namdo, Republic of Korea; 2 University of Ulsan College of Medicine, Seoul, Seoul-t’ukpyolsi, Republic of Korea; 3 Asan Medical Center, Songpa-gu, Seoul-t’ukpyolsi, Republic of Korea

## Abstract

**Background:**

The optimal duration of antimicrobial therapy for uncomplicated *Pseudomonas aeruginosa* bloodstream infection (BSI) is unknown. We compared the outcomes of short and prolonged courses of antimicrobial therapy in adults with uncomplicated pseudomonal BSI.

**Methods:**

All patients with uncomplicated *P. aeruginosa* BSI admitted at a tertiary-care hospital from May 2016 to September 2020 were included. We compared the rate of recurrent *P. aeruginosa* infection and 30-day mortality among patients who underwent short (7‒11 days) and prolonged (12‒21 days) courses of antimicrobial therapy using propensity score analysis with the inverse probability of treatment weighting (IPTW) method.

**Results:**

We evaluated 1,477 patients with uncomplicated *P. aeruginosa* BSI; of them, 290 met the eligibility criteria, including 97 (33%) who underwent short-course therapy (9 [interquartile range (IQR), 8‒11] days) and 193 (67%) who underwent prolonged-course therapy (15 [IQR, 14‒18] days). We found no significant difference in the risk of recurrence or 30-day mortality between the prolonged-course and short-course groups (n=10, 11% vs. n=32, 16%; IPTW-adjusted hazard ratio (HR) 0.61; 95% confidence interval (CI) 0.30−1.24; *p*=0.17). The recurrence of *P. aeruginosa* infection at any site within 180 days of completing therapy occurred significantly more in the prolonged-course group (n=10, 10% vs. n=38, 20%; IPTW-adjusted HR 0.48; 95% CI 0.24−0.96, *p*=0.04). The resistance acquisition in subsequent *P. aeruginosa* isolates was more frequent in the prolonged-course group, although the difference was not statistically significant (n=2, 20% vs. n=12, 32%; *p*=0.70).

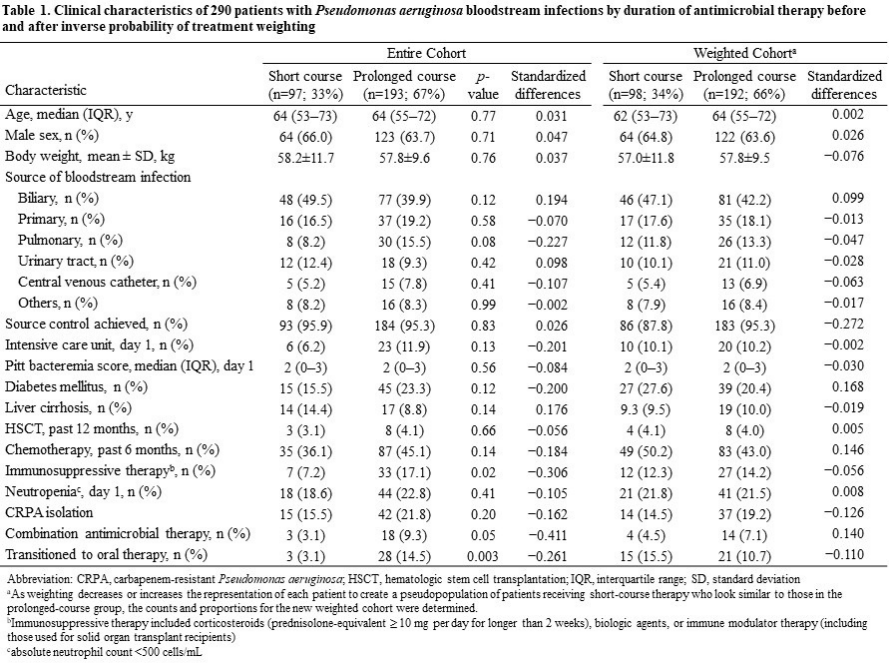

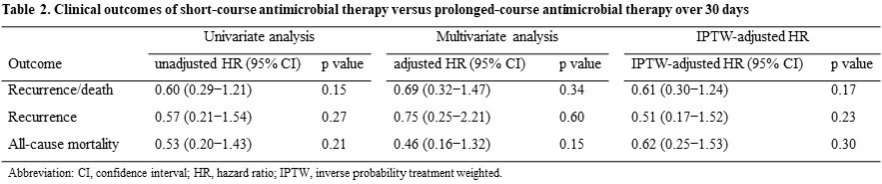

**Conclusion:**

Short-course antimicrobial therapy could be as effective as prolonged-course therapy for uncomplicated *P. aeruginosa* bloodstream infection.

**Disclosures:**

**All Authors**: No reported disclosures

